# Genome-Wide Association Studies Provide Insight Into the Genetic Determination for Hyperpigmentation of the Visceral Peritoneum in Broilers

**DOI:** 10.3389/fgene.2022.820297

**Published:** 2022-03-01

**Authors:** Guangyuan Zhou, Tianfei Liu, Yan Wang, Hao Qu, Dingming Shu, Xinzheng Jia, Chenglong Luo

**Affiliations:** ^1^ Guangdong Provincial Key Laboratory of Animal Molecular Design and Precise Breeding, School of Life Sciences and Engineering, Foshan University, Foshan, China; ^2^ State Key Laboratory of Livestock and Poultry Breeding, Guangdong Key Laboratory of Animal Breeding and Nutrition, Institute of Animal Science, Guangdong Academy of Agricultural Sciences, Guangzhou, China

**Keywords:** genome-wide association studies, hyperpigmentation, chicken, carcass traits, high-throughput sequencing

## Abstract

Hyperpigmentation of the visceral peritoneum (HVP) has been becoming one of the most challenging problems in yellow-feathered chicken production, which seriously affected chicken carcass quality traits. Detecting which genes dominantly impact pigmentation in the peritoneum tissues is of great benefit to the genetic improvement of HVP. To investigate the genetic mechanism of HVP in yellow-feathered broilers, genome-wide association studies (GWASs) were conducted in the F_2_ generation of a cross broiler population with 395 birds. A total of 115,706 single-nucleotide polymorphisms (SNPs) of 122,415 were retained to identify quantitative trait loci (QTL) associated to HVP in chicken. The GWAS results based on the logistic mixed model (LMM) revealed that a narrow genomic location on chromosomes 1 (49.2–51.3 Mb) was significantly associated (*p* ≤ 4.32 × 10^−7^) with HVP, which contained 23 SNP makers related to 14 functional genes (*MFNG*, *POLDIP3*, *POLR2F*, *PICK1*, *PDXP*, *SGSM3*, *RANGAP1*, *MYH9*, *RPL3*, *GALP3*, *LGALS1*, *MICALL1*, *ATF4*, and *CYP2D6*). Four highly associated (*p* < 10^−5^) haplotype blocks of 0.80 kb (two SNPs), 0.06 kb (two SNPs), 0.95 kb (two SNPs), and 0.03 kb (two SNPs) were identified with two, two, four, and four distinct haplotypes, respectively. As a melanoma-associated gene, *CYP2D6* were also possibly involved in the development of HVP occurring in chicken with two significant variations (rs314284996 and rs317955795) in the promoter regions. Further tests revealed that the expression of *CYP2D6* was obviously higher in the visceral peritoneum tissue of chicken with HVP than that in the normal group (*p* < 0.05). Our results provide a novel clue to understand the genetic mechanism of HVP generation in chicken, and the mapped QTL or candidate genes might serve for genomic selection to improve carcass quality in the yellow-feathered chicken industry.

## Introduction

**FIGURE 1:**
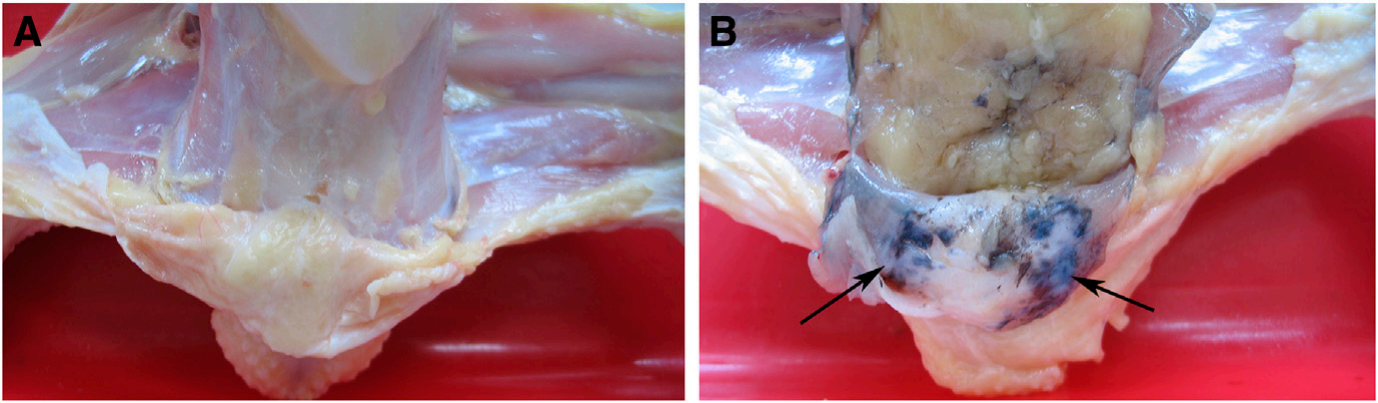
Visceral peritoneum of the normal chicken **(A)** and HVP chicken **(B)**.

All breeds of domestic fowl may have hyperpigmentation in the skin of the shanks and abdominal fascia, resulting in a decline in the appearance of the carcass (Crespo and Pizarro, 2006). Hyperpigmentation of the visceral peritoneum (HVP) has been becoming one of the most important problems impacting carcass quality in the commercial yellow-feathered chicken industry in China, with up to 90% occurs in some indigenous breeds, causing serious economic losses to the broiler production (Wang et al., 2014). To some extent, the generation of HVP was similar to fibromelanosis. However, the pigmentation dominantly occurred in chicken peritoneum tissues with HVP issues, resulting in an abnormal navy-blue skin color (Figure 1). The genetic causes of HVP occurring are still unknown. It was possibly caused by melanocytes producing the pigment melanin, similar to Silkie chicken peritoneum melanin (Wang et al., 2014).

Genome-wide association studies (GWASs) were proved to be a viable and successful approach to explore phenotype-to-genotype connections (Visscher et al., 2017), which have been widely utilized in livestock and poultry breeding (Yang et al., 2015; Tian et al., 2020). Currently, a large amount of candidate genes were identified as causal markers associated with growth performance, meat quality, carcass traits, feather colors, and disease resistance ability in the Chinese indigenous chicken population (Luo et al., 2014; Zhang et al., 2015; Mao et al., 2019; Guo et al., 2020; Zhang et al., 2020). For example, GWAS in an F_2_ resource population was conducted to identify two novel candidate genes related to three single-nucleotide polymorphisms (SNPs) associated with avian influenza antibody titers (Sun et al., 2016). Another study reported that 21 candidate genes were proved to be associated with chicken bone growth based on GWAS in an F_2_ crossbred experimental population (Li et al., 2021). For the HVP performance, our previous studies have preliminary detected three highly associated QTL regions (GGA1:50.5–54.0 Mb, GGA1:58.5–60.5 Mb, and GGA20:10.5–12.0 Mb) using Illumina Chicken 60 K SNP Beadchip in an F_2_ cross broiler population (Luo et al., 2013).

When melanin formed in melanocytes is abnormally produced, skin hyperpigmentation disorder develops (Roselan et al., 2020). Dermal hyperpigmentation, also known as fibromelanosis (FM), is a typical skin pigmentation trait in chickens, and FM is linked to higher *EDN3* expression (Arora et al., 2011). However, the *EDN3* gene may not be a crucial gene for the black phenotype in sheep, which is marked by black pigmentation throughout the body and on internal organs (Darwish et al., 2018). Lentigines and freckles are common skin hyperpigmentations in humans. The quantity of melanocytes in lentigines and freckles differs. The number of melanocytes in freckles remains constant, but the amount of melanin produced increases; on the other hand, lentigines are caused by a rise in the number of melanocytes (Kumari et al., 2018).

In this study, a fine-mapping GWAS based on the F_2_ resource population was conducted to explore the genetic variations or genome segments controlling HVP occurring in Chinese yellow chicken. The candidate genes and markers associated with HVP can help elucidate the genetic architecture of carcass traits involved in hyperpigmentation distribution and can subsequently be used for marker-assisted selection or genomic selection in poultry breeding.

## Materials and Methods

### Ethical Statement

The animal experiments were performed with the approval of the Animal Care Committee of the Institute of Animal Science, Guangdong Academy of Agricultural Sciences (Guangzhou, China) and Use Committee (GAAS-IAS-2009-73).

### Experimental Animals

The experimental population was a three-generation cross-breeding population construction from two different meat-type chicken lines. The first founder line was Huiyang Bearded Chicken (HB), which is an indigenous breed in China. The second founder line was the commercial broiler breed “High Quality Chicken A Line” (HQLA). The ratio of HVP of Huiyang Bearded Chicken is much higher than that of HQLA. A total of 395 individuals from the F_2_ population were used for GWAS, including 212 cocks and 183 hens from eight half-sib families. At 91 days of age, venous blood was collected and slaughtered. As shown in Figure 1, HVP is evaluated by peeling: chickens with visceral peritoneum pigmentation are HVP chickens, and chickens with no peritoneal pigmentation are normal chickens. Among the 395 birds, 124 individuals had HVP and 271 individuals had absent HVP. Heritability estimation was carried out by GAPIT (Wang and Zhang, 2021) software. We used a linear mixed model to estimate genetic variance based on genomic information.

### Genotyping and Quality Control

**FIGURE 2:**
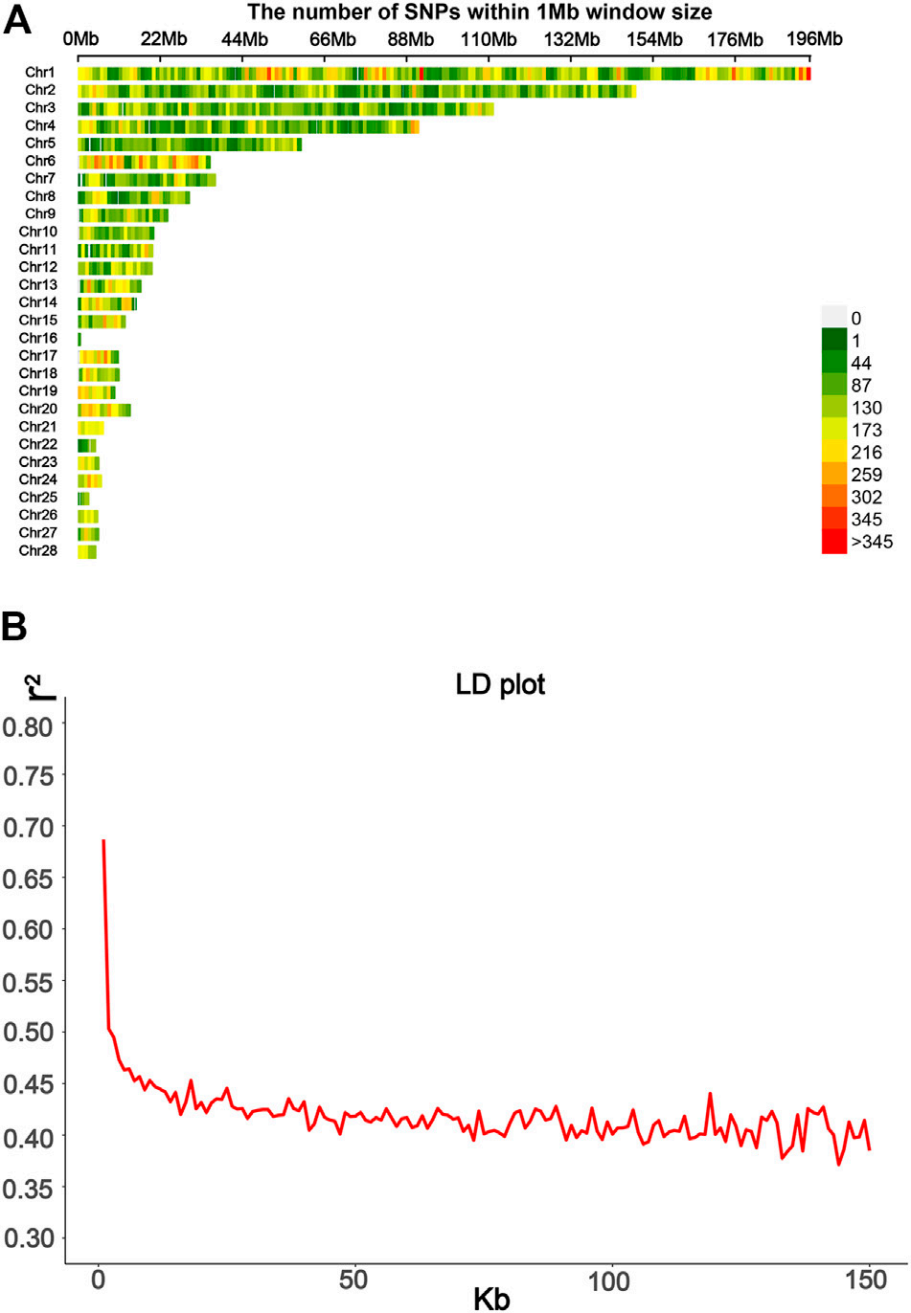
Information from SLAF-seq genotyping was used in this study. **(A)** SNPs’ density distribution on 28 autosomes; the SNP density based on a 1 Mbp window was estimated. **(B)** Average distance between SNPs was used to calculate the LD decay plot.

Genomic DNA was extracted from venous blood using the phenol/chloroform method as described before (Jia et al., 2016). All the birds were genotyped using the 10X SLAF-seq (Specific-Locus Amplified Fragment Sequencing), which was performed by Biomarker Technologies (Beijing, China) Co., Ltd. For enzyme cutting site prediction, the genome of Gallus gallus 5.0 (ftp://ftp.ncbi.nlm.nih.gov/genomes/all/GCF/000/002/315/GCF_000002315.4_Gallus_gallus-5.0) was used as the reference genome. The qualifying DNA of the sampled individuals was digested using HaeIII restriction enzymes. The length of paired-end reads was 125 bp, while the sequencing insert size was 364–444 bp. The consistent part of the variation obtained by GATK (DePristo et al., 2011) and samtools (Li et al., 2009) was used as the final variation site for subsequent analysis. After quality control, 115,706 SNP markers remained with call rate >70% and MAF> 0.01. BEAGLE version 5.2 was used to impute missing SNPs (Browning and Browning, 2013). The 30X SLAF-seq data were obtained from 22 F_0_, with individuals as a reference panel. *r* ^2^ of LD was calculated using PLINK 2.0 (Chang et al., 2015); then, regrouping was done, and the mean of LD and the LD decay plot were obtained using the ggplot2 package. The SNP distribution is shown in Figure 2A. The LD decay line quickly decreased first; after 50 kb, the distance tended to be slow (Figure 2B). These values thus indicate that the F_2_ population has relatively short LD distances and quick LD decay.

### Statistical Analysis

GWAS was performed using the logistic mixed model (LMM); the statistical model is

$$logit\left(\pi_{i}\right)=X_{i}\alpha +G_{i}\beta +b_{i}$$

where $\pi_{i}=P\left(y_{i}=1\right)$ is the probability of a HVP for animal I; X_i_ is the vector of covariates for animal i; *α* is the vector of fixed covariate effects including the intercept, gender, batch, and two principal components (PC1 and PC2); Gi is the genotype of animal i; and β is the genotype effect. It was assumed that $b\;∼\;N\left(0,\;\sigma_{g}^{2}K\right)$ , where $\sigma_{g}^{2}$ is the additive genetic variance and **K** is the additive genetic relationship matrix based on genomic information. GWAS was implemented in the GMMAT (Chen et al., 2016) package, which is based on the R language.

The Bonferroni correction (Benjamini and Hochberg, 1995) was used to control the false positive rate in the GWAS analyses, namely, *p* = 0.05/N, where N is the number of SNP loci. In this study, we used a genome-wide significance threshold *p*-value of 4.32*10^−7^ (i.e., *p* = 0.05/115,706). Quantile–quantile (QQ) plots and Manhattan plots were plotted using the R package CMplot (Yin et al., 2021). Use of a web-based platform ensemble (URL:http://archive.ensembl.org/Gallus_gallus/Info/Index) translated the significant SNPs from Gallus gallus 5.0 to Gallus gallus 6.0.

The population structure was analyzed using Admixture software (Alexander et al., 2009), and admixture proportions were visualized with the R package pophelper (Francis, 2017). The genomic inflation factor (λ), defined as the ratio of the genome-wide chi-squared statistics mean (or median) to the expected distribution of this statistic under the null hypothesis, was used to assess the impact of population stratification on association analyses. Generally, the more the value of genomic inflation factor deviates from 1, the higher the rates of false positive errors in GWAS analyses (Devlin et al., 2001). The formula for the genomic inflation factor is as follows:

$$\lambda =\frac{Median\left(T_{i}^{2}\right)}{0.4549}$$

where $Median\left(T_{i}^{2}\right)$ is the median of the statistic from the real data and 0.4549 is the expected median under the null hypothesis.

Linkage disequilibrium (LD) of SNP loci significantly associated with HVP was performed with the software haploview (Barrett et al., 2005). After the haplotypes significantly associated with HVP are identified, candidate genes in the haplotype blocks are detected. The statistical analysis of haplotypes with HVP was performed with the R package haplo.stats (Schaid et al., 2002).

### Gene Identification and Annotation

The nearest genes present within 50 kb upstream or 50 kb downstream of the SNP loci significantly associated with HVP were identified as potential candidate genes based on the Gallus gallus 6.0 reference genome (http://archive.ensembl.org/Gallus_gallus/Info/Index).

### Quantity of mRNA Expression by qRT-PCR

The visceral peritoneum tissues were collected from adult chicken with extremely different HVP performance with no peritoneal pigmentation (*n* = 5) (absent HVP control group) and five HVP-positive (severe HVP) chickens. The total RNA was extracted with RNA TRIzol (Lin et al., 2019) reagents according to standard protocols. An M-MLV RTase cDNA Synthesis Kit was used to prepare cDNA. The visceral peritoneum *MFNG*, *MYH9*, *RANGAP1*, and *CYP2D6* gene expression was determined by quantitative real-time polymerase chain reaction (qRT–PCR). qRT–PCR primers were designed using NCBI Primer-Blast. The reactions of qRT–PCR were performed in 20 µL volumes. Parameters of RT-PCR were as follows: 3 min 95°C, followed by 40 cycles of 30 s at 95°C, 30 s at 60°C, and 45 s at 72°C. The relative gene expression levels of the target genes *MFNG*, *MYH9*, *RANGAP1*, and *CYP2D6* were calculated using the *2* ^*−△△CT*^ method and normalized with housekeeping genes: *GAPDH*. The differential expression of *MFNG, MYH9*, *RANGAP1*, and *CYP2D6* in the visceral peritoneum tissue, between the normal and HVP-positive, was determined using a *t*-test with the software Graphpad Prism 8 (Knapek et al., 2020).

## Results

### Population Structure Analysis

**FIGURE 3:**
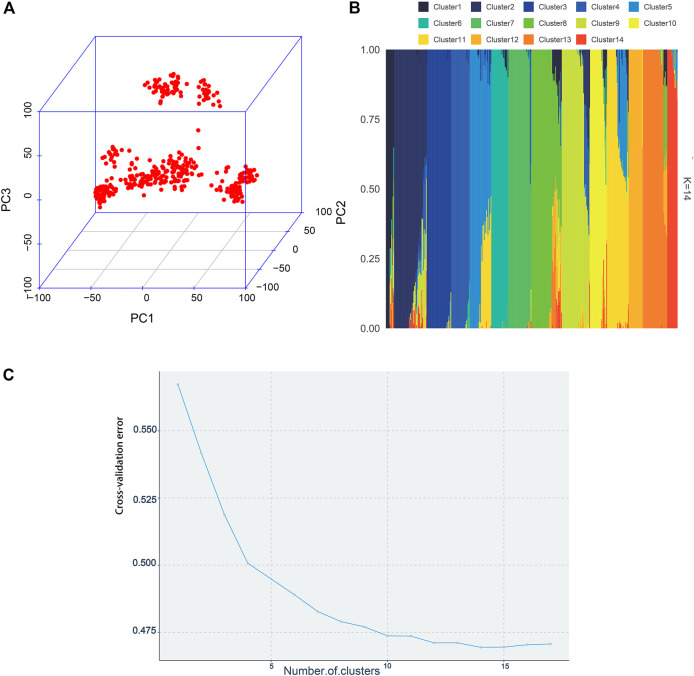
Population structure plots demonstrated by the 395 individuals. **(A)** By constructing the 3D plot, the first three PCs were chosen to depict the population structure. **(B)** Admixture analysis. Each bar represents an individual. Different colors represent various genetic clusters, and the proportion of the color represents the likelihood of an individual being allocated to that genetic cluster. The individuals were sorted by aligning clusters using the alink function of R package pophelper. **(C)** Cross-validation errors across 17 ancestral genetic clusters.

The visualization of population stratification was performed by principal component analysis (PCA) for the first three principal components (PCs) (Figure 3). The first two PCs, gender, and batch were used as covariates in the GWAS to avoid the false positive caused by population stratification. The genomic inflation factor of the logistic mixed model is 1.006, indicating that the rates of false positive errors are well controlled. The decay of LD was the strongest within the first 50 kb (Figure 2B). These SNPs corresponding to the nearest genes within the LD decay range were selected for identification of the candidates.

### GWAS on HVP Trait

**FIGURE 4:**
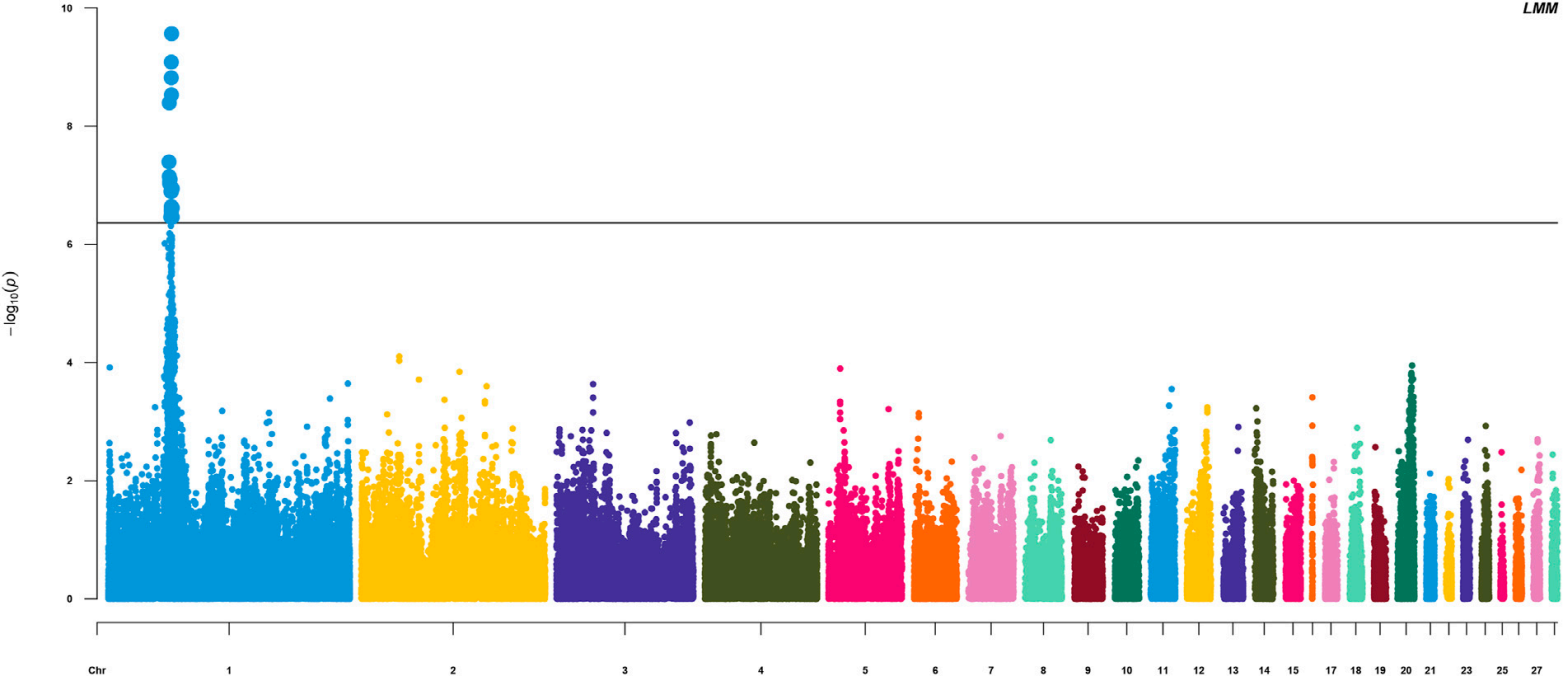
Manhattan plots for analyzing the results of HVP. Significant SNP loci are indicated in bold.

The HVP heritability was 0.32 in this F_2_ population of yellow chicken, which illustrated that the HVP trait could be potentially improved by selective breeding. GWAS performed on HVP showed that 23 consistent SNPs were identified as the most significantly associated loci with HVP performance, which was analyzed by the logistic mixed model (Figure 4). Meanwhile, these SNPs were all from a narrow genomic location on chromosomes 1 (49.2–51.8 Mb). To some extent, the results were partly overlapped with our previous study (Luo et al., 2013).

**FIGURE 5:**
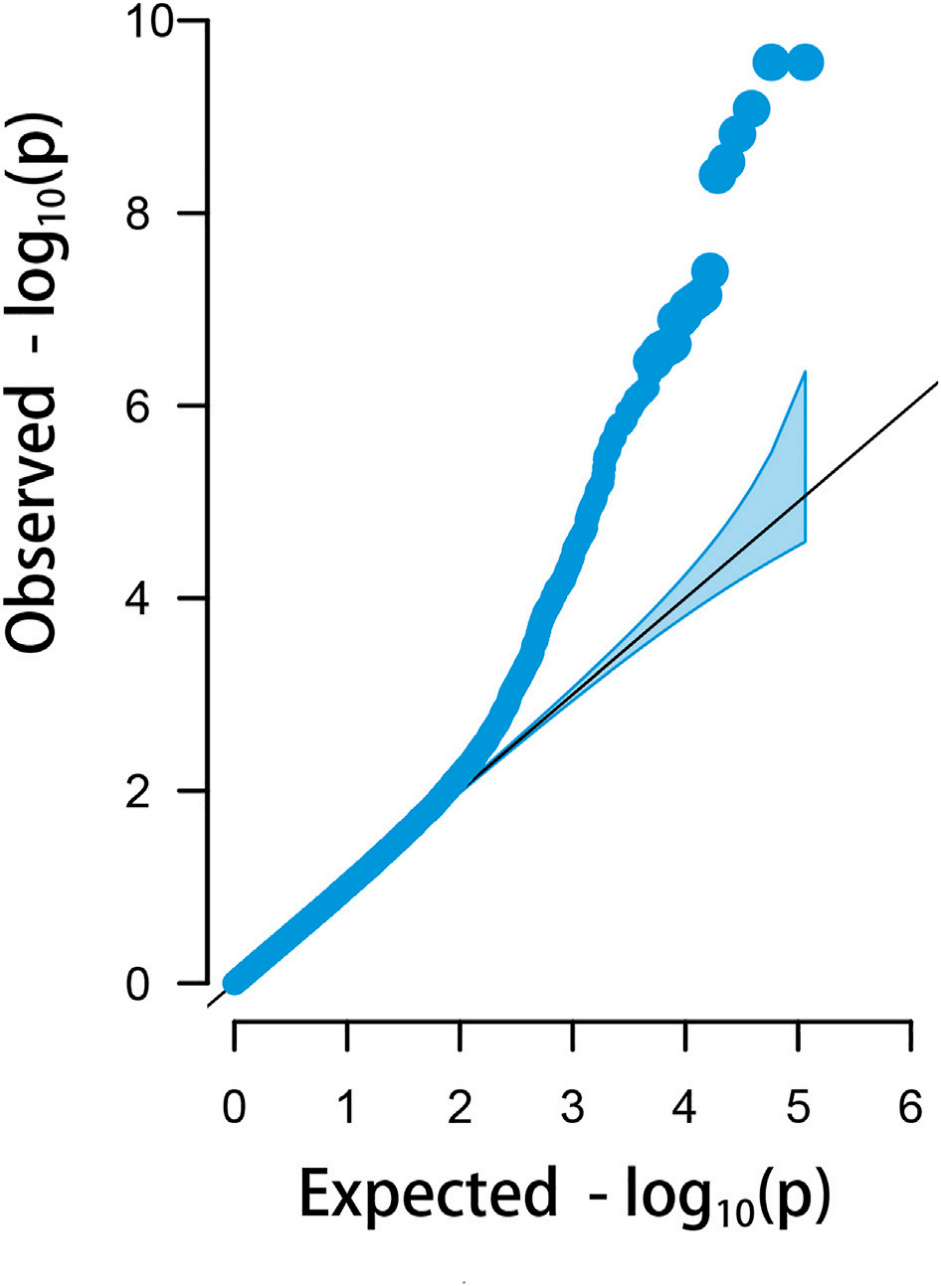
Quantile–quantile plots of the logistic mixed model (LMM) analyzed for HVP traits in China yellow chickens.

There were 23 potential candidate genes located in this region, including *MFNG*, *POLDIP3*, *POLR2F*, *PICK1*, *PDXP*, *SGSM3*, *RANGAP1*, *MYH9*, *RPL3*, *GALP3*, *LGALS1*, *MICALL1*, *ATF4*, and *CYP2D6* (Table 1). Of which, rs314284996 and rs317955795 were located in the promoter regions of *CYP2D6* (less than 1.3 kb distance from 5′UTR). Interestingly, *CYP2D6* was identified as a biomarker associated with increased susceptibility to melanoma, which is coded for another enzyme that may play a role in detoxifying potentially carcinogenic compounds (Bose et al., 2018). A quantile–quantile plot of the logistic mixed model analyzed for HVP traits showed that the observed and expected *p*-values of the GWAS fitted very well (Figure 5).

**TABLE 1:** Significant SNPs with *p* ≤ 4.32 × 10^−7^ from GWAS.

SNP	Chr	Position (bp)	Nearest gene^a^	Distance (Kb)^b^	*p*-value
rs736467172	1	51282572	MFNG	D5.96	2.71E-10
rs741188868	1	51282630	MFNG	D6.02	2.71E-10
rs737774287	1	51070638	POLR2F	within	5.23E-10
rs1058585183	1	51021678	PICK1	within	1.51E-09
rs734622509	1	51176674	PDXP	U5.02	2.94E-09
rs314284996	1	49407107	CYP2D6	U1.29	4.03E-09
rs317933544	1	49182509	POLDIP3	U0.96	4.02E-08
rs317955795	1	49407909	CYP2D6	U0.49 within U0.79	7.12E-08
rs734376222	1	50080245	SGSM3	U0.70	7.92E-08
rs317439499	1	49733538	***RANGAP1***	D20.40	8.64E-08
rs732579815	1	49733625	***RANGAP1***	U11.25 within within D20.38	9.38E-08
rs735088380	1	51840959	***MYH9***	U6.81	1.14E-07
rs80653259	1	50708200	RPL3	U1.69 within within within U2.10 within	1.27E-07
rs737034753	1	51117437	GALP3		1.28E-07
rs732895613	1	51067727	POLR2F		2.29E-07
rs317658316	1	51840929	***MYH9***		2.42E-07
rs740218724	1	51159699	LGALS1		2.47E-07
rs317243218	1	51253923	MFNG		2.60E-07
rs313652047	1	51067889	POLR2F		2.62E-07
rs736902992	1	51092656	MICALL1		2.81E-07
rs13652709	1	51067809	POLR2F		3.13E-07
rs736693927	1	50597253	ATF4		3.44E-07
rs13866298	1	51816134	MYH9		3.46E-07

a Genes in bold italics have been detected in the previous study by Luo et al. (2013).

b U means that the SNP is in the upstream of the gene, and D means that the SNP is in the downstream of the gene.

### Haplotype-Based Associated Analysis on HVP Traits

**FIGURE 6:**
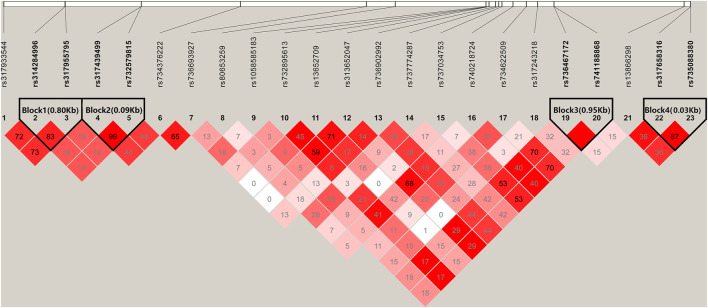
Linkage disequilibrium analysis of significant SNPs. Values in boxes are LD (*r* ^2^) between SNP pairs. The absence of the *r* ^2^ value within the boxes means *r* ^2^ = 1, complete linkage disequilibrium (LD). The LD color display is based on the D′/LOD score ratio.

There were four haplotype blocks, which spanned 0.80, 0.09, 0.06, and 0.03 kb genomic regions (Table 2), and they were identified to have significant association with HVP traits (Figure 6), with a conservative LD threshold (*r* ^2^ = 0.8). As shown in Table 3, the haplotypic Block 1 included two significant SNPs, with only two SNP haplotypes constructed. The AG haplotype has a significant minus effect on HVP (*p* < 0.01), and the GT haplotype has a great plus effect on HVP (*p* < 0.01). However, a total of two haplotypes were generated in Block 2, and the two haplotypes were significantly associated with HVP. The AT haplotype has a significant minus effect on HVP (*p* < 0.01), and the GC haplotype has a great plus effect on HVP (*p* < 0.01). The haplotypic Block 3 included four significant SNPs, with only two SNP haplotypes constructed. The TG haplotype has a significant minus effect on HVP (*p* < 0.01), and the CA haplotype displayed a plus effect on HVP (*p* < 0.01). However, a total of four haplotypes were generated in Block 4 with two haplotypes significantly associated with HVP traits. The CT haplotype has a significant minus effect on HVP (*p* < 0.01), and the TC haplotype has a great plus effect on HVP (*p* < 0.01).

**TABLE 2:** Significant SNPs in the LD Block.

LD block	Size (Kb)	SNP	Location	Nearest gene	Distance (Kb)^a^
Block1	0.80	rs314284996	49407107	CYP2D6	U1.32
		rs317955795	49407909	CYP2D6	U0.52
Block2	0.09	rs317439499	49733538	RANGAP1	U0.79
		rs732579815	49733625	RANGAP1	U0.70
Block3	0.06	rs736467172	51282572	MFNG	D16.06
		rs741188868	51282630	MFNG	D16.12
Block4	0.03	rs317658316	51840929	MYH9	D20.38
		rs735088380	51840959	MYH9	D20.40

a U means that the SNP is in the upstream of the gene, and D means that the SNP is in the downstream of the gene.

**TABLE 3:** Results of haplotype association analysis.

Block	Haplotype	Haplotype frequency	Haplotype score	*p*-Value	Global *P* value^a^
Block1	AG	0.62405	−7.36153	≤10^−5^	≤10^−5^
	GT	0.37595	7.36153	≤10^−5^	
Block2	AT	0.51013	−5.54528	≤10^−5^	≤10^−5^
	GC	0.48861	5.54528	≤10^−5^	
Block3	TG	0.76177	−6.31563	≤10^−5^	≤10^−5^
	TA	0.01797	−0.74768	0.45466	
	CG	0.01291	1.31051	0.19002	
	CA	0.20734	6.59693	≤10^−5^	
Block4	CT	0.7277	−6.03254	≤10^−5^	≤10^−5^
	CC	0.01028	0.09231	0.92645	
	TT	0.13559	0.9448	0.34476	
	TC	0.12643	7.09266	≤10^−5^	

a Global *p*-value: *p*-value of score.global based on Chi-square distribution, with degrees of freedom equal to df.

### Analysis of the Expression Patterns of HVP Candidate Genes

**FIGURE 7:**
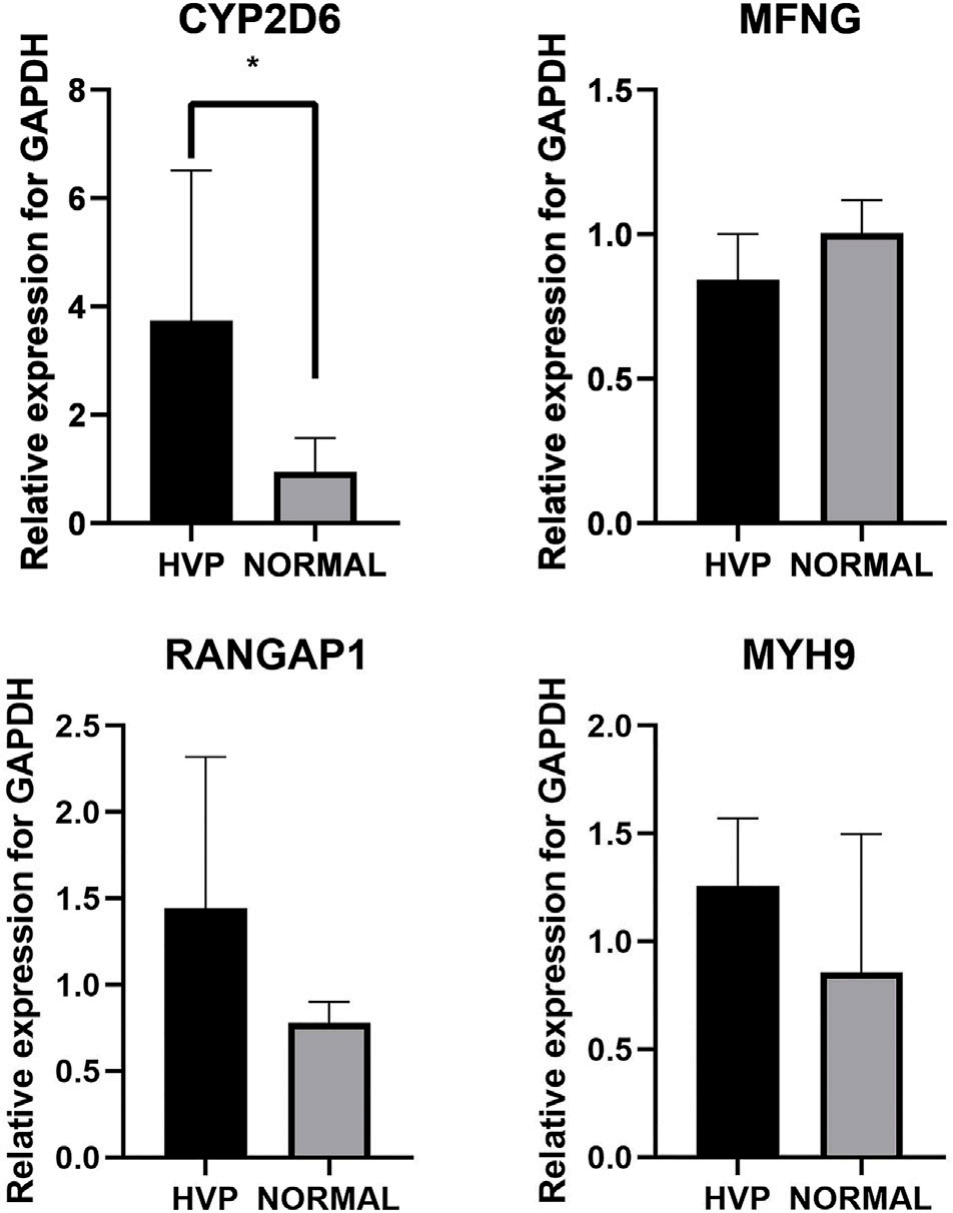
Expression of chickens *CYP2D6*, *MYH9*, *RANGAP1*, and *MFNG*; * indicates significant differences at *p* < 0.05.

Exploring expression level changes in candidate genes was proved to be helpful to identify major genes following GWAS analysis. Here, five birds were randomly chosen from both the severe HVP and absent HVP groups and were utilized for detecting transcriptional expression profiles of *CYP2D6*, *MYH9*, *RANGAP1*, and *MFNG* by qRT-PCR. The primers are listed in Table 4. The results showed that both these functional genes were expressed in the visceral peritoneum tissues (Figure 7). Only the *CYP2D6* gene was greatly differentially expressed between the birds from the absent and severe HVP groups (*p* < 0.05), and *CYP2D6* had a much high level in the server individuals, which was consistent with the studies in humans that *CYP2D6* genotypes were associated with the hair color, Breslow thickness, and malignant melanoma (Strange et al., 1999). For the *MFNG* gene, coding an oncogene through Notch-mediated signaling (Kakuda et al., 2020), there was no statistically significant difference in birds with various HVP performances (*p* > 0.05). For the *MYH9* gene, which encodes the non-muscle myosin heavy-chain IIA protein (Pecci et al., 2018), there was no statistically significant difference in birds with various HVP performances (*p* > 0.05). For the *RANGAP1* gene, a crucial component of the RanGTPase system (Lin et al., 2016), there was no statistically significant difference in birds with various HVP performances (*p* > 0.05).

**TABLE 4:** Primer sequences for qRT-PCR.

Gene	Forward primer 5->3′	Reverse primer 5->3′
*CYP2D6*	AAC​TGT​GAA​GGA​AGC​CCT​GG	CTT​GCC​AAA​ACA​AGC​CCT​TCA
*MFNG*	GGG​AAC​CTG​ACT​CTT​GGG​GA	ACC​ACA​TGG​TCA​CCC​ATT​CTC
*MYH9*	GCT​GAT​TAG​ACA​AGT​GCG​GG	AGT​TTG​CGG​ACG​TGC​TTG​AT
*RANGAP1*	AGT​CGG​AGC​TCA​AGA​GGT​GT	AAA​GGC​GTT​GTC​ACT​CAG​GT
*GAPDH*	GGT​GAA​AGT​CGG​AGT​CAA​CGG	TCG​ATG​AAG​GGA​TCA​TTG​ATG​GC

## Discussion

HVP is one of the most important carcass traits in the production of chilled carcass broilers, which made it difficult to carry out breeding selection at the early age of yellow-feathered chickens in China with a longer production cycle. However, our studies showed that HVP has a medium heritability in the F_2_ cross population, indicating that it is possible to improve HVP performance through molecular breeding selection with the precious understanding knowledge of the genetic mechanism on this trait. A recent study found that designed populations, such as F_2_ populations for GWASs, were very beneficial in enhancing QTL mapping accuracy (Ledur et al., 2010). Designed GWAS on the F_2_ population presented great power to identify the causal genetic markers and genes controlling complex economic traits. Based on GWAS investigation, a narrow QTL on chromosome 1 was identified to have significant genomic architectures associated with HVP traits. Joint analysis between GWAS and gene expression patterns showed that *CYP2D6* was a potential genetic marker for breeding selection on HVP. Our results provided novel clues to understand the genetic mechanism of HVP generation in chicken and improved conduction of genomic selection on HVP traits in the yellow-feathered chicken industry.

The broiler chicken population used in this study was constructed by crossing a Huiyang Beard chicken and a Chinese commercial broiler breed. Huiyang Beard chickens are one of the indigenous Chinese chicken breeds. There were several problems for breeding in most local breeds, such as smaller effective population numbers, which resulted in lower diversity in breeds and a higher LD level and larger fraction of SNPs with fixed alleles (Munoz et al., 2019). However, there was a clear genetic diversity among these indigenous Chinese chicken breeds such as in Beard chickens, Chahua chickens, and Silkie and Langshan chickens (Yuan et al., 2018). The LD analysis (Figure 2B) showed a small distance of LD decay in this F_2_ population, which could be helpful for QTL mapping on HVP traits in our study with high genetic diversity. Our GWAS results proved very clear associated signals between a narrow QTL and HVP performance (*p* ≤ 4.32 × 10^−7^).

Improvements in the density of SNPs at the whole genome level have allowed more accuracy in mapping resolution of common variant associations in GWAS. Based on the same broiler population, Liu et al. (2020) revealed that the accuracy of genomic prediction using SLAF-seq was higher than that using SNP chips. These results indicate that SLAF-seq covers more important SNPs with effects. As the marker density increases, the accuracy of QTN identification in GWAS also increases (Liu et al., 2018). The previous study by Luo et al. (2013) used a medium-density chip with only 46 K SNPs, while SLAF-seq has 115 K SNPs in the current study, and the distributions of the two types of markers are different. The use of more markers is expected to detect more genes that associate with HVP. The aim of the present study is to find new candidate genes for HVP based on SLAF-seq. The results in the current study validated part of the results of the previous study and detected 11 new candidate genes for HVP, and joint analysis of haplotype association and gene expression pattern changes indicated that CYP2D6 was a novel potential gene for HVP. It suggested that the SNP density has great power to improve QTL mapping, and even twice the size of SNP number was obtained in this study.

Fine-mapping analysis indicated that the correlation between gene expression patterns and genotypes based on GWAS QTL mapping has more accuracy to identify QTNs regulating complex traits in livestock (Liu et al., 2018). A large number of GWASs also proved that combining multiple approaches had a great advantage in improved QTL identification and interpretation (Abed and Belzile, 2019). Here, based on GWAS results with the LMM model, 23 significant SNPs were discovered at the first step. Then, haplotype-based association analysis was employed to narrow down effectiveness of taking into account the linkage disequilibrium (Epstein and Satten, 2003), resulting in four significant haplotype blocks related to *CYP2D6*, *MYH9*, *RANGAP1*, and *MFNG*. Finally, the gene expression pattern between case-control groups was utilized to explore the candidate genes. *CYP2D6* was identified as a major candidate gene for HVP by integrating all these tests. Moreover, the functional of *CYP2D6* also contributed to our conclusion. *CYP2D6* encodes a highly polymorphic gene in the family of cytochrome P450, and its function is highly variable (Li et al., 2019). Strange et al. (1999) revealed that there was an association between *CYP2D6* genotypes with tumor thickness in human cutaneous melanoma, and polymorphisms in *CYP2D6* are associated with hair color. In melanoma, *CYP2D6* was thought to be related to prognosis and risk (Howell, 2004). *CYP2D6* genetic polymorphism may provide an entry point to explore propranolol in the management of melanoma (De Giorgi et al., 2018; Patane, 2018). Sun et al. (2019) analyzed the RNA-seq data in melanoma from TCGA (The Cancer Genome Atlas) database through the Kyoto Encyclopedia of Genes and Genomes (KEGG) pathway enrichment analyses, and CYP2D6 was found to be enriched in the cytochrome P450 pathway. Therefore, either GWAS or molecular function annotation indicated that *CYP2D6* could be a novel genetic marker for HVP performance in chickens.

As the sequencing technology improves, the possibility of the causal mutations being incorporated in the genomic selection model increases. However, needless to say, more variant diversity was much better for genomic selection (Broeckx et al., 2017; Jung et al., 2020). The significantly associated loci discovered by GWAS can be used to construct genomic prediction models and achieve genetic gains through genomic selection (Sehgal et al., 2015; McGowan et al., 2020). GWAS-assisted genomic selection has yielded promising results in several research studies. In a simulation study, when the genomic association matrix was weighted on the causative mutations, the results showed a 167% improvement in prediction accuracy (Fragomeni et al., 2017). In real data analysis, the genomic prediction models integrating two or more GWAS markers as fixed effects can substantially improve the accuracy (Odilbekov et al., 2019). When *meta*-GWAS significantly associated loci were added as fixed effect factors in GS models assessing height in Jersey and Holstein bulls, the prediction accuracies increased (Raymond et al., 2018). Therefore, our study is useful for marker-assisted selection or genomic selection in poultry breeding.

## Conclusion

In this study, GWAS in the F_2_ cross broiler population was conducted for investigating the genetic basis of HVP traits based on a proper genotyping by SLAF-seq. A narrow QTL region of 2.1 Mb (49.2–51.3 Mb) on chromosome 1 (GGA1) was identified clearly associated with HVP, related to 23 significantly associated SNPs. Gene annotations revealed that 14 genes (*MFNG*, *POLDIP3*, *POLR2F*, *PICK1*, *PDXP*, *SGSM3*, *RANGAP1*, *MYH9*, *RPL3*, *GALP3*, *LGALS1*, *MICALL1*, *ATF4*, and *CYP2D6*) could be primary candidate genes for HVP occurring in chicken. Joint analysis of haplotype association and gene expression pattern changes indicated that *CYP2D6* was a novel potential genetic marker for HVP performance in chicken with much higher expression in the birds of severe HVP, which was in agreement with previous studies in humans that *CYP2D6* genotypes were highly associated with tumor thickness in cutaneous melanoma. Our results provide a new insight to understand the genetic basis of HVP generation in chicken and improve the accuracy of genomic selection for carcass quality in the poultry breeding.

## Data Availability

The original contributions presented in the study are included in the article/Supplementary Material, and further inquiries can be directed to the corresponding authors.
