# Supplementary figures and images for: Genome-Wide Association Studies Provide Insight Into the Genetic Determination for Hyperpigmentation of the Visceral Peritoneum in Broilers
Source: Front Genet. 2022 Mar 1;13:820297. doi: 10.3389/fgene.2022.820297 (PMC8921551; doi:10.3389/fgene.2022.820297)

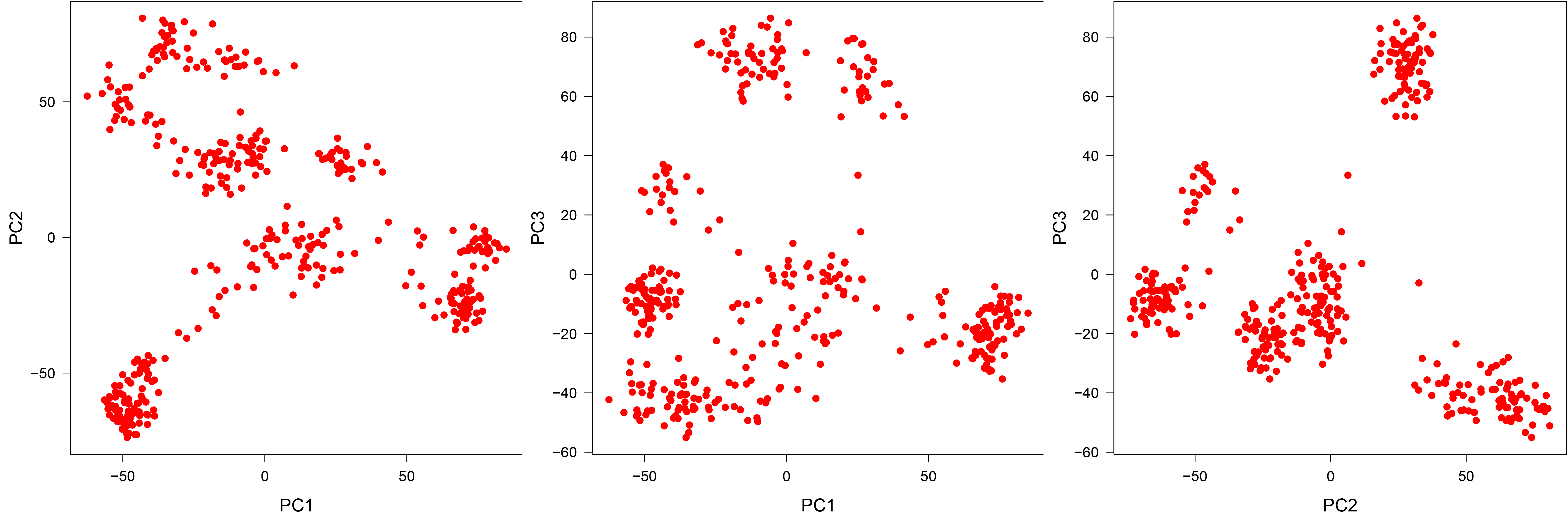

Supplement: Supplementary file 1 [file Image1.JPEG]
