# Supplementary material for: Genome-Wide Association Studies Provide Insight Into the Genetic Determination for Hyperpigmentation of the Visceral Peritoneum in Broilers
Source: Front Genet. 2022 Mar 1;13:820297. doi: 10.3389/fgene.2022.820297 (PMC8921551; doi:10.3389/fgene.2022.820297)
Supplement: Supplementary file 2 [file DataSheet1.docx]

Supplementary TABLE 1 |

| **half-sib families** | **NORMAL** | | **HVP** |  |
| --- | --- | --- | --- | --- |
| A | 7 | 2 | | |
| B | 13 | 0 | | |
| C | 18 | 5 | | |
| D | 65 | 18 | | |
| E | 42 | 49 | | |
| F | 11 | 0 | | |
| G | 64 | 23 | | |
| H | 51 | 27 | | |

Supplementary FIGURE 1 | The population structure plots. The three highest principal components (PCs) were chosen to show the population structure by building the 2D graphic.
